# Astrocyte expression of the *Drosophila* TNF-alpha homologue, Eiger, regulates sleep in flies

**DOI:** 10.1371/journal.pgen.1007724

**Published:** 2018-10-31

**Authors:** William M. Vanderheyden, Alan G. Goodman, Rebecca H. Taylor, Marcos G. Frank, Hans P. A. Van Dongen, Jason R. Gerstner

**Affiliations:** 1 Elson S. Floyd College of Medicine, Washington State University, Spokane, Washington, United States of America; 2 Sleep and Performance Research Center, Washington State University, Spokane, Washington, United States of America; 3 School of Molecular Biosciences and Paul G. Allen School of Global Animal Health, College of Veterinary Medicine, Washington State University, Pullman, Washington, United States of America; Katholieke Universiteit Leuven, BELGIUM

## Abstract

Sleep contributes to cognitive functioning and is sufficient to alter brain morphology and function. However, mechanisms underlying sleep regulation remain poorly understood. In mammals, tumor necrosis factor-alpha (TNFα) is known to regulate sleep, and cytokine expression may represent an evolutionarily ancient mechanism in sleep regulation. Here we show that the *Drosophila* TNFα homologue, Eiger, mediates sleep in flies. We show that knockdown of Eiger in astrocytes, but not in neurons, significantly reduces sleep duration, and total loss-of-function reduces the homeostatic response to sleep loss. In addition, we show that neuronal, but not astrocyte, expression of the TNFα receptor superfamily member, Wengen, is necessary for sleep deprivation-induced homeostatic response and for mediating increases in sleep in response to human TNFα. These data identify a novel astrocyte-to-neuron signaling mechanism in the regulation of sleep homeostasis and show that the *Drosophila* cytokine, Eiger, represents an evolutionarily conserved mechanism of sleep regulation across phylogeny.

## Author summary

Every animal sleeps, from fruit flies to humans. However, the function of sleep is still currently unknown. Identifying conserved mechanisms of sleep regulation in evolutionarily ancient organisms may help us to understand the function of sleep. Therefore, we have examined whether Eiger, the homologue of the cytokine tumor necrosis factor-alpha (TNFα), regulates sleep in the fruit fly as it does in higher mammals. Cytokines are inflammatory molecules and are typically elevated following infection or fever and may contribute to increased sleepiness when sick. We found that, in the fruit fly, Eiger regulates sleep duration just like TNFα does in mammals: increasing cytokine levels increased sleep duration while decreasing Eiger reduced sleep. In addition, we found that Eiger expression in glial astrocytes, is responsible for the alteration in sleep duration. We also examined the necessity of Eiger receptor activation on neurons and found that astrocyte-to-neuron communication was required for regulating the normal increases in sleep following sleep deprivation. These data show that a novel cytokine mechanism regulates sleep in flies and mammals, and provides insight into conserved roles of astrocytes in sleep behavior.

## Introduction

The function of sleep and the neurobiology underlying the detrimental effects of sleep deprivation on physiological function are poorly understood. Understanding phylogenetic conservation of mechanisms that regulate sleep and the neurobiological consequences associated with sleep loss may provide important clues to sleep function. Although the function of sleep is unknown, sleep is regulated by a combination of genetic and experience-dependent environmental influences. For example, environmental conditions such as temperature, light levels, and social interactions are sufficient to modify sleep duration or sleep architecture [1]. Additionally, many genes have been found to play a role in the regulation of sleep [2–6]. Specifically, in the fruit fly *Drosophila melanogaster*, molecules involved in inflammation and immunological functioning have been implicated in sleep regulation including nuclear factor kappa-light-chain-enhancer of activated B cells (NF-kB) [7] and Jun amino-terminal kinases (JNK) [8]. In mammals, another such molecule is the pro-inflammatory cytokine, tumor necrosis factor alpha (TNFα). TNFα functions as a sleep regulatory substance [9,10]. TNFα promotes non-REM (NREM) sleep when administered centrally or peripherally [9,11], and neural activity [12] and sleep deprivation [13] both increase production of TNFα. TNFα is also an important regulator of homeostatic synaptic scaling [14] and experience-dependent plasticity [15]. TNFα signaling has been implicated in cognitive impairment due to sleep deprivation in humans [16], and in many human diseases including cancers [17], depression [18], and neurodegenerative disorders like Alzheimer’s disease [19–21]. Many of these diseases are associated with sleep abnormalities and impaired cognition. Given the importance TNFα signaling for sleep in mammals and the role of inflammation and immunological genes in sleep regulation in the fly, we examined the *Drosophila melanogaster* TNFα homologue, Eiger, to determine evolutionarily conserved mechanisms of cytokines in sleep.

Eiger was first discovered in a p-element screen to examine cell death mechanisms [22] and was the first cytokine to be discovered in the fruit fly [23]. The majority of the work on Eiger has focused on cell death, apoptosis, infection, and JNK signaling pathways. Here, we assessed behavioral and molecular properties of sleep and Eiger expression in subpopulations of fruit fly central nervous system (CNS) cells in order to determine the role of Eiger and the Eiger receptor, Wengen, in the regulation of sleep duration and sleep architecture. The data presented here suggest that Eiger underlies phylogenetically conserved mechanism of sleep regulation and identifies a unique astrocyte-to-neuron mechanism regulating sleep behavior.

The fly is an ideal organism to examine phylogenetically conserved mechanisms of sleep regulation. The genetics of the fly are easily manipulated and sleep in the fruit fly shares many of the features of mammalian sleep [24,25]. Specifically, sleep in the fruit fly is dependent upon waking experience and homeostatically regulated. Similar to mammals, flies show an increase in sleep following prolonged periods of sleep deprivation [4,5,26,27]. Additionally, courtship conditioning and social enrichment both result in increased sleep [28]. Learning tasks increase the expression of plasticity-related molecules and increase sleep duration [1,29] and similar observations have been made in mammalian systems [30,31]. In mammals, when a brain area is activated during wake, cytokine expression increases [32,33] and the subsequent EEG delta power in that brain area increases [31,34,35]. Taken together, these data suggest that the fly may be an important model to test for evolutionarily conserved mechanisms of cytokine dependent sleep regulation.

## Results

### Human TNF alpha increases sleep in *Drosophila*

**Fig 1:**
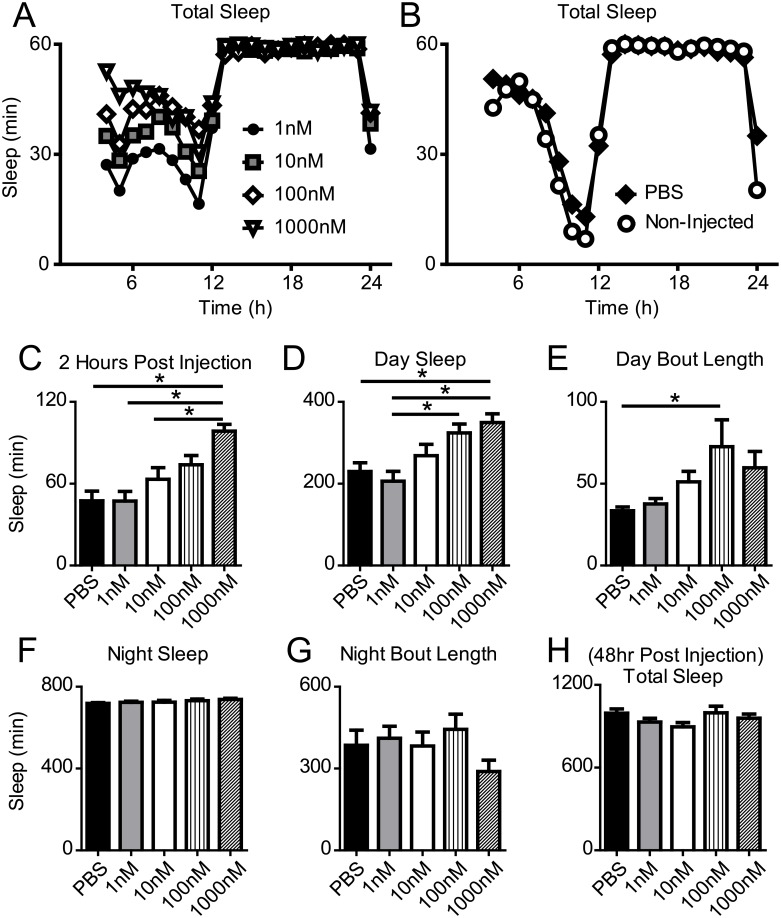
Recombinant human TNFα increases sleep in wild-type flies. (A) Sleep profiles plotted for flies injected with recombinant human TNFα at ZT4 (concentrations varied from 1-1000nM). (B) Sleep profile of PBS injected and non-injected controls; vehicle injection did not significantly alter baseline sleep duration compared to non-injected controls (t(56) = 1.465, p = 0.149). (C) Sleep duration increased in the 2 hours immediately following TNFα injection in a dose-dependent manner. One-way ANOVA shwed a significant main effect of TNFα injection (F(4,138) = 9.377, p < 0.0001). Tukey post-hoc test revealed that 1000nM TNFα concentration (n = 29, M = 98.6, SD = 26.7) resulted in significantly increased sleep compared to PBS controls (n = 23, M = 47.5, SD = 34.0), 1nM TNFα injection (n = 30, M = 47.3, SD = 39.0), and 10nM TNFα injection (n = 29, M = 63.3, SD = 45.4). The 100nM concentration (n = 28, M = 73.8, SD = 36.2) did not differ from the 1000nM concentration. (D) Total remaining daytime sleep was significantly increased in the 8 hours after TNFα injection in a similar dose-response manner (from PBS (M = 229.9, SD = 102.2) to 1nM (M = 206.2, SD = 131.6), 10nM (M = 268.4, SD = 149.1), 100nM (M = 324.2, SD = 113.6), and 1000nM (M = 349.6, SD = 114.0)) (one-way ANOVA, F(4,138) = 6.786, p < 0.0001). (E) Sleep bout length during the daytime was increased by injections of TNFα (one-way ANOVA, F(4,138) = 2.811, p = 0.028). (F-G) On the day of injection, subsequent nighttime sleep duration (F, (F(4,138) = 1.24, p = 0.30) and bout length (G, (F(4,138) = 1.42, p = 0.23) were unaffected by TNFα. (H) The effects of TNFα were short-lived and did not affect long term sleep behavior. Total sleep, 48 hours after the injections, was unchanged by injection of TNFα (F(4,108) = 1.62, p = 0.175).

Eiger shares significant homology to human TNFα and TNFα superfamily members [22]. To examine if human TNFα is sufficient to increase sleep in *Drosophila* similarly to its effect in mammals [34], we performed intra-thoracic injections of recombinant human TNFα into wild-type flies and measured sleep for 3 days. Sleep duration increased with increasing concentrations of TNFα (Fig 1A). Whereas PBS injected controls did not show significantly different sleep profiles from non-injected controls (Fig 1B). Specifically, total sleep duration was significantly increased in a dose-response manner in the 2 hours immediately following injection (Fig 1C) and in the subsequent 8 daytime hours (Fig 1D). TNFα injections (100nM) also resulted in a significant increase in the length of daytime sleep bouts (Fig 1E). Nighttime sleep bout duration (Fig 1F) and bout length (Fig 1G) were unaffected by injection of TNFα. 48 hours after injection, total sleep duration in all TNFα treated groups was not significantly different from PBS injected controls (Fig 1H). These data indicate that the injection resulted in an acute change in sleep duration and there were no long-term consequences of the varied concentrations of TNFα on sleep duration.

### Eiger loss-of-function mutants show reduced sleep duration

**Fig 2:**
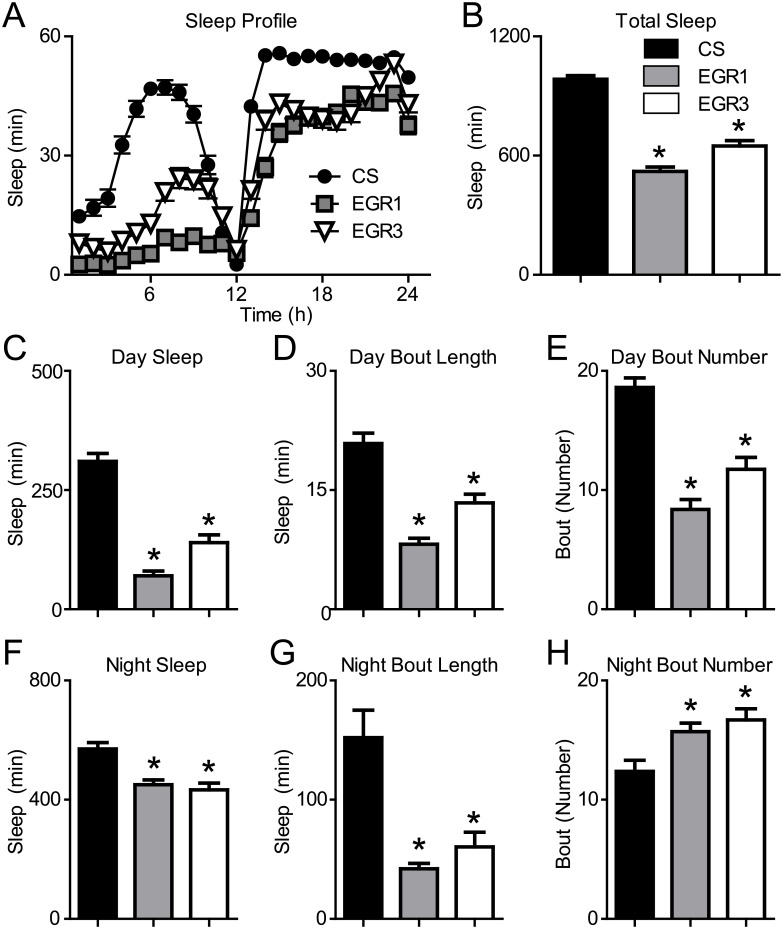
Eiger loss-of-function mutants show reduced baseline sleep. (A,B) The Eiger 1 (EGR1) (n = 95, M = 520.3, SD = 214.6) and Eiger 3 (EGR3) mutants (n = 83, M = 648.5, SD = 247.2) showed a significant reduction in total sleep duration compared to wild-type Canton S (CS) control flies (n = 85, M = 984.0, SD = 173.8) (one-way ANOVA, F(2,262) = 110.7, p < 0.0001). (C) Daytime sleep duration was significantly reduced in Eiger mutants EGR1 (M = 70.1, SD = 99.5), EGR3 (M = 158.4, SD = 158.3), versus CS (M = 345.6, SD = 133.6) (one-way ANOVA, F(2,262) = 102.30, p < 0.0001). (D) Daytime bout length was significantly reduced in Eiger mutants: EGR1 (M = 8.2, SD = 6.9), EGR3 (M = 13.4, SD = 9.9), versus CS (M = 20.9, SD = 11.9) (One-way ANOVA, F(2,262) = 36.05, p < 0.0001)). (E) Daytime bout number was significantly reduced in Eiger mutants: EGR1 (M = 8.38, SD = 7.70), EGR3 (M = 11.75, SD = 8.85), versus CS (M = 18.61, SD = 7.27) (one-way ANOVA, F(2,262) = 36.41, p < 0.0001)). (F) Eiger mutants showed reduced nighttime sleep duration: EGR1 (M = 450.3, SD = 157.9), EGR3 (M = 490.3, SD = 164.2), versus CS (M = 637.6, SD = 73.8) (one-way ANOVA, F(2,262) = 44.26, p < 0.0001)) (G) Eiger mutants showed reduced nighttime bout lengths: EGR1 (M = 42.1, SD = 42.9), EGR3 (M = 60.4, SD = 112.3), vesus CS (M = 152.2, SD = 211.9) (one-way ANOVA, F(2,262) = 15.86, p < 0.0001))). (H) The number of sleep bouts during the night was significantly increased in the Eiger mutants: EGR1 (M = 15.7, SD = 7.0), EGR3 (M = 16.6, SD = 8.6), versus CS (M = 12.3, SD = 8.7) (one-way ANOVA, F(2,262) = 6.64, p = 0.0015)). Asterisks indicate values that are significantly different (p < 0.05) from CS control flies by Tukey’s Multiple Comparison Test.

TNFα is a potent sleep regulatory substance in mammals. TNFα levels correlate positively with slow wave sleep and sleep duration [36] and inhibition of TNFα reduces sleep duration in rats and rabbits [37,38]. To determine the role of Eiger in sleep regulation in the fly, we tested Eiger 1 (EGR1) and Eiger 3 (EGR3) loss-of-function mutants and examined the necessity of Eiger in regulating baseline sleep. Both Eiger mutants displayed a reduction in total sleep duration (Fig 2A and 2B) compared to wild-type Canton S (CS) control flies. During the day (Fig 2C–2E) and the night (Fig 2F–2H), the EGR1 and EGR3 mutants showed decreased sleep duration (Fig 2C and 2F) and reduced sleep bout length (Fig 2D and 2G). The number of sleep bouts was reduced during the day (Fig 2E) and increased during the night (Fig 2H) in both mutant lines compared to control flies. During the night, a reduction of sleep bout length along with an increase in the number of sleep bouts suggests that these flies experience significant nighttime sleep fragmentation. These data are in agreement with the mammalian literature, which suggests that inhibition of TNFα is sufficient to reduce sleep.

**Fig 3:**
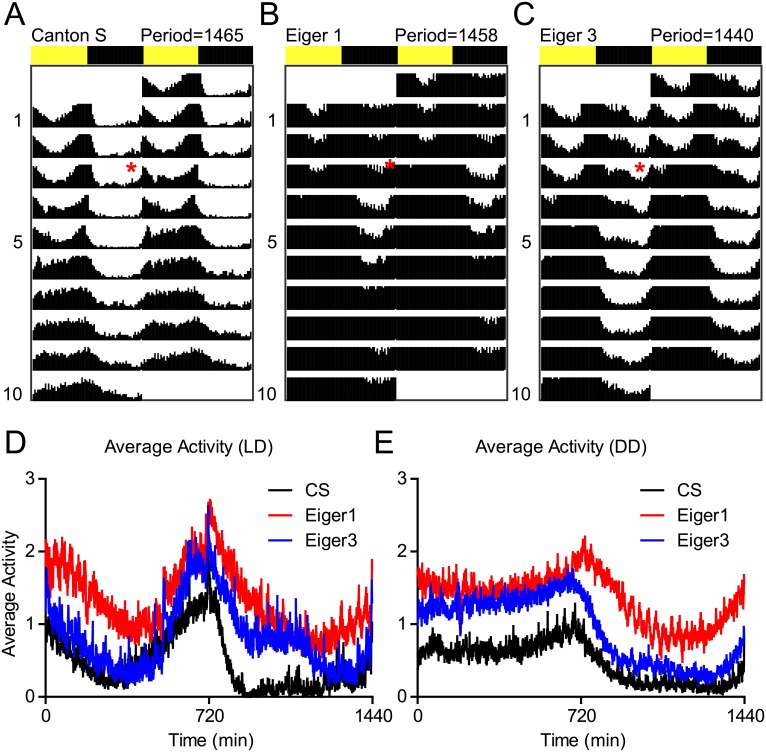
Eiger loss of function results in periods of hyperactivity, but does not alter circadian function. (A-C) Actograms for flies exposed to 4 days of 12:12, Light:Dark cycle before being placed into 12:12, Dark:Dark (red asterisks) to measure baseline activity and circadian periodicity. Activity counts are shown on double plotted actograms for 10 days (top to bottom). Circadian period was calculated using the ActogramJ plugin for ImageJ for CS (n = 32), Eiger1 (n = 32), and Eiger3 mutants (n = 32). Eiger mutants did not show a significantly altered circadian period from wild-type CS flies. (D,E) The ActogramJ plugin was additionally used to show average activity levels as measured by beam breaks in the Trikinetics monitoring system during the Light:Dark and Dark:Dark portions of the circadian experiment.

To assess the effect of the EGR1 and EGR3 mutations on circadian function and behavioral activity levels, we examined groups of CS, EGR1, and EGR3 flies during 4 days on a 12:12, Light:Dark cycle and then transitioned all flies to Dark:Dark (Fig 3A–3C, red star indicates transition to Dark:Dark). Then, using the *ActogramJ* Plugin for ImageJ, we generated double plotted actograms and circadian periods were calculated. None of the three lines tested, including the Eiger mutant lines, showed significantly impaired circadian rhythms during Dark:Dark. Additionally, we plotted the average daily locomotor activity during the Light:Dark period and Dark:Dark periods (Fig 3D and 3E) and found that the Eiger mutants were hyperactive at different times of the day compared to CS control flies during the Light:Dark (Fig 3D) and Dark:Dark (Fig 3E) periods. These activity data show that the active periods in the Eiger mutants correspond to the timing of reduced sleep shown in Fig 2.

### Injection of human TNFα increases sleep in Eiger loss-of-function mutants

**Fig 4:**
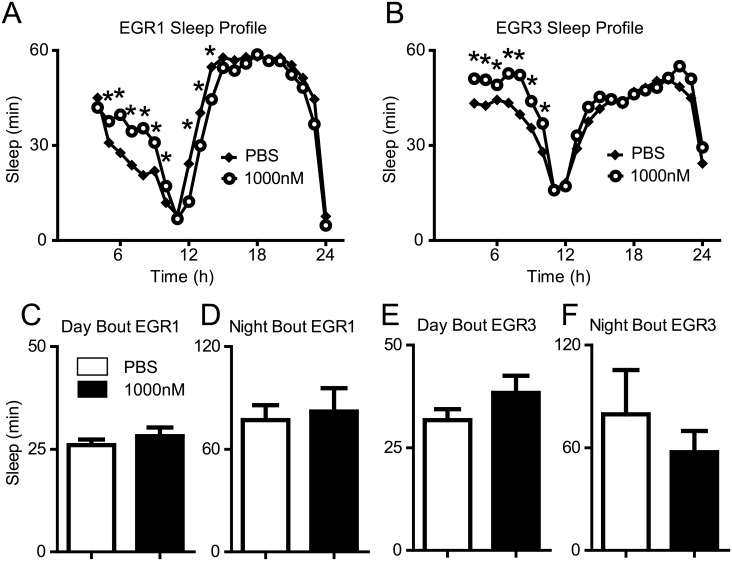
Injection of human TNFα increases sleep in Eiger mutants. (A,B) Baseline sleep profiles are shown for EGR1 mutants (A) and EGR3 mutants (B) injected with vehicle (PBS) or 1000nM recombinant TNFα. Two-way ANOVA revealed a significant time by drug interaction for the EGR1 mutants (F(1,20) = 91.49, p < 0.0001) and for the EGR3 mutants (F(1,20) = 18.74, p < 0.0001). Tukey Post Hoc analysis was used to identify significant changes in sleep between PBS and TNFα injected flies (* = p < 0.05). (C-F) Student’s t-tests revealed that the increase in sleep was not associated with an increase in bout length during the day or night in either genotype (p > 0.05).

Human TNFα is sufficient to increase sleep in wild-type flies (see above), however, it is unknown whether TNFα injections would have the same effect in short-sleeping Eiger mutant flies. To examine if human TNFα is sufficient to increase sleep in the Eiger mutants similarly to wild-type flies, we performed TNFα injections on the EGR1 and EGR3 mutants. Injection of 1000nM human TNFα was sufficient to increase sleep in both the EGR1 (Fig 4A) and EGR3 (Fig 4B) mutants. The increase in sleep duration was not associated with an increase in average bout length during the day or the night for either mutant line (Fig 4C–4F).

### Eiger RNAi knockdown in astrocytes reduces sleep duration

**Fig 5:**
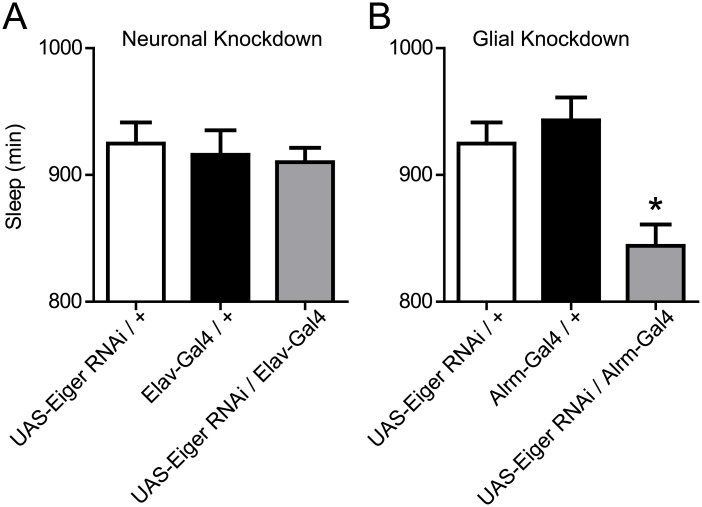
Astrocytes contribute to Eiger-mediated reduction in sleep duration. (A) RNAi knockdown of Eiger in neurons using the Elav-Gal4 driver (n = 126, M = 910.1, SD = 126.7) did not alter total sleep duration compared to Elav-Gal4/+ (n = 119, M = 915.8, SD = 212.1) and UAS-Eiger RNAi/ + (n = 116, M = 924.8, SD = 179.6) parental controls F(2,360) = 0.214, p = 0.81). (B) RNAi knockdown of Eiger in astrocytes using the Alrm-Gal4 driver (n = 91, M = 844.1, SD = 160.7) significantly reduced total sleep duration compared to Alrm-Gal4/+ (n = 90, M = 943.1, SD = 171.1) and UAS-Eiger RNAi/ + (n = 116, M = 924.8, SD = 179.6) parental controls F(2,296) = 8.735, p = 0.0002). Tukey post-hoc tests revealed that total sleep in the Eiger RNAi / Alrm-Gal4 flies (grey bar) was significantly reduced when compared to both the Eiger RNAi/+ (p < 0.005) and Alrm-Gal4/+ (p < 0.005) parental lines.

In mammalian brains, TNFα is produced in neurons and glia [14,39]; in the fly, Eiger is enriched in astrocytes [40]. To determine whether the Eiger loss-of-function sleep duration phenotype is confined to a particular cell type, we used a previously validated UAS-Eiger RNA-interference (RNAi) line [22] to knockdown Eiger expression in neurons (using Elav-Gal4) versus astrocytes (using Alrm-Gal4). We assessed sleep duration in these knockdown lines compared to their corresponding control parental lines. RNAi knockdown in neurons had no effect on total sleep duration (Fig 5A), while astrocyte knockdown significantly reduced total sleep duration (Fig 5B).

### Neuronal expression of the Drosophila TNFα receptor Wengen is required for normal sleep homeostasis

**Fig 6:**
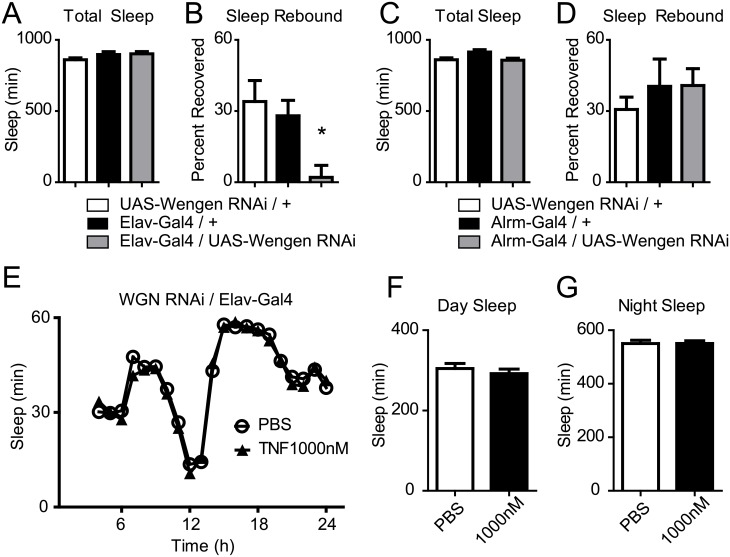
Effects of RNAi knockdown of the Eiger receptor, Wengen. (A-D) Baseline sleep and the homeostatic response to sleep loss were measured in flies where UAS-Wengen RNAi was expressed in neurons (A,B) or astrocytes (C,D). Baseline total sleep duration for flies expressing neuronal UAS-Wengen RNAi (grey bar) (A, F(2,373) = 1.77, p = 0.17) or astrocyte UAS-Wengen RNAi (C, F(2,85) = 1.87, p = 0.16) was not significantly changed compared to parental controls (white and black bars). (B) The homeostatic response was reduced for flies expressing neuronal UAS-Wengen RNAi, (F(2,88) = 5.95, p = 0.0038). (D) The homeostatic response was unchanged when UAS-Wengen RNAi was expressed in astrocytes (D, F(2,130) = 0.599, p = 0.55) compared to parental controls. (E) Baseline sleep profile for UAS-Wengen RNAi/Elav-Gal4 expressing flies injected with either PBS (n = 58), or 1000nM TNFα (n = 61). (F-G) Total day sleep duration (F, t(117) = 0.76, p = 0.45) and night sleep duration (G, (t(117) = 0.0241, p = 0.98) were unchanged by TNFα injections.

Wengen was the first TNFα receptor superfamily member found in *Drosophila*, and it plays a critical role in transducing Eiger mediated cell death signals [41]. To further elucidate the role of Eiger in sleep regulation, we explored the cell-type specificity of the Eiger receptor, Wengen, in regulating baseline sleep and sleep homeostasis. We expressed the previously validated UAS-Wengen RNAi [41] in neurons (Fig 6A and 6B) and astrocytes (Fig 6C and 6D) using the same Gal4 drivers as presented in Fig 5 (Elav-Gal4, neurons; Alrm-Gal4, astrocytes). Baseline sleep was unaffected by neuronal (Fig 6A) and astrocyte driven (Fig 6C) Wengen-RNAi expression. Following 12 hours of sleep deprivation, neuronal RNAi-meditated knockdown of Wengen showed a significant reduction in sleep rebound compared to control flies (Fig 6B), while RNAi-mediated knockdown of Wengen in astrocytes showed a normal homeostatic response (Fig 6D). This neuronal knockdown of Wengen resulted in a similar sleep homeostasis phenotype as Eiger loss-of-function mutants (S1 Fig), pointing to an astrocyte-to-neuron signaling pathway via Eiger and Wengen, which may be a critical regulator of the homeostatic response to sleep deprivation.

To determine whether neuronal Wengen is necessary for TNFα effects on regulating sleep duration or architecture in *Drosophila*, as in Figs 1 and 4, we injected 1000nM human recombinant TNFα (or vehicle) in UAS-WGN RNAi/ Elav-Gal4 flies and measured changes in baseline sleep. TNFα failed to alter baseline sleep in neuronal expressing Wengen RNAi flies (Fig 6E–6G). These data suggest that neuronal expression of Wengen is required for TNFα induced changes in sleep.

### Eiger is required for experience-dependent increases in sleep duration

**Fig 7:**
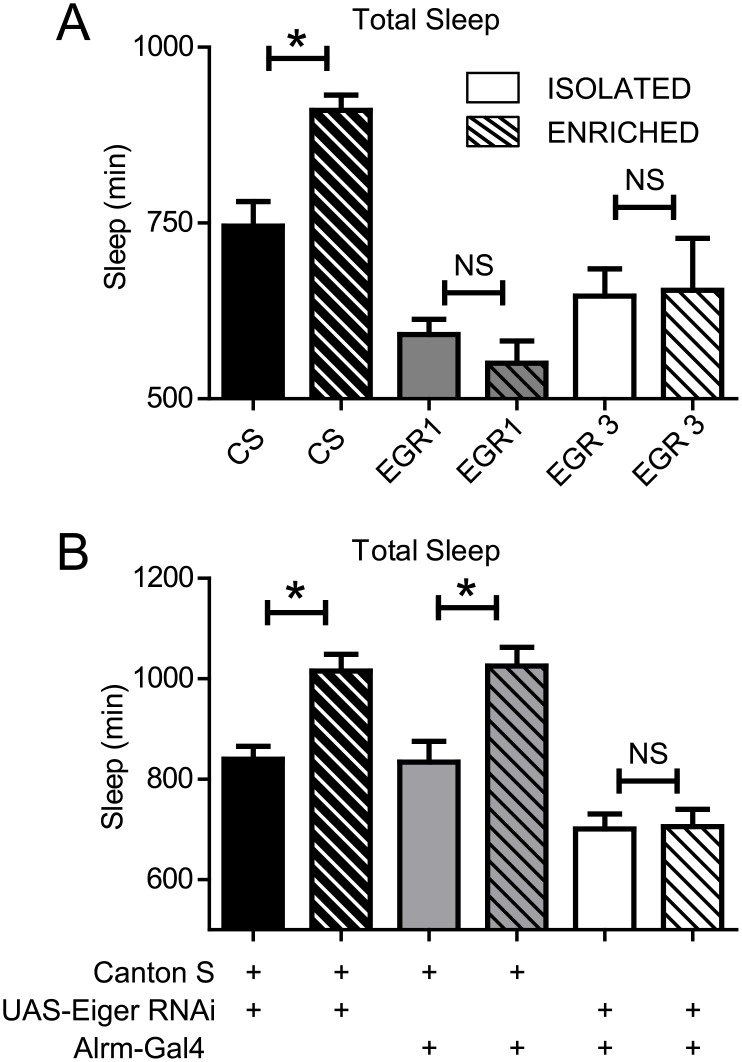
Social enrichment-induced increases in sleep are dependent upon Eiger expression. (A) 5 days of social enrichment in wild-type CS flies increased total sleep duration (n = 29, M = 910.1, SD = 117.3) compared to socially isolated siblings (n = 31, M = 732.1, SD = 184.9). However, neither the EGR1 mutants (enriched n = 30, M = 550.5, SD = 174.1; isolated, n = 30, M = 591.5, SD = 119.4) nor the EGR3 mutants (enriched n = 29, M = 654.3, SD = 398.0; isolated, n = 30, M = 646.2, SD = 211.7) showed experience-dependent changes in sleep. One-way ANOVA revealed a significant effect of enrichment F(5,178) = 9.9, p < 0.0001); asterisks indicate values that are significantly different (p < 0.05) from isolated flies as determined by Tukey’s Multiple Comparison Test. (B) RNAi knockdown of Eiger in astrocytes prevented the increase in experience-dependent changes in sleep (enriched n = 32, M = 705.7, SD = 195.1; isolated, n = 32, M = 701.2, SD = 167.0) while parental control lines (RNAi/+ in black (enriched n = 32, M = 1015.0, SD = 189.9; isolated, n = 28, M = 839.8, SD = 135.5); Alrm-Gal4/+ in grey (enriched n = 31, M = 1025.0, SD = 210.2; isolated, n = 30, M = 834.1, SD = 224.4)) both showed a significant increase in sleep in response to social enrichment. One-way ANOVA revealed a significant effect of enrichment F(5,184) = 17.8, p < 0.0001); asterisks indicate values that are significantly different (p < 0.05) from isolated flies as determined by Tukey’s Multiple Comparison Test.

Social experience (i.e. enrichment) increases sleep duration in *Drosophila*. This may reflect interactions between synaptic plasticity and sleep need. Given that TNFα mediates experience-dependent plasticity in mammals [15], and social experience increases sleep duration in flies [1,28,42], we next investigated whether Eiger also mediated experience-dependent changes in sleep duration. We exposed CS, EGR1, and EGR3 flies to either social enrichment or social isolation for 5 days and then measured subsequent changes in sleep. Following 5 days of social enrichment, wild-type CS flies showed the typical increase in sleep (M = 178 minutes, P<0.05), whereas, neither EGR1 (M = -41 minutes, P>0.05) nor EGR3 (M = 8 minutes, P>0.05) mutants increased sleep (Fig 7A) compared to their isolated counterparts.

To assess whether astrocytes contribute to this enrichment phenotype, we expressed Eiger-RNAi in astrocytes and performed the same social enrichment paradigm as above. The parental lines for the Eiger-RNAi/+ and Alrm Gal4/+ flies both showed an increase in sleep in response to social enrichment (black and grey bars in Fig 7B). However, expression of the Eiger-RNAi construct in astrocytes was sufficient to block experience-dependent increases in sleep (Fig 7B).

## Discussion

We show that human TNFα is sufficient to increase sleep in the fruit fly, similar to what is seen in mammals [9,11]. Conversely, Eiger loss-of-function using the EGR1 and EGR3 mutants resulted in a significant reduction in total sleep; a similar phenotype as seen in mammals when TNFα is inhibited [37,38]. These data suggest that TNFα and Eiger share functional homology across species. Additionally, we showed cell-type specificity of Eiger signaling in these sleep phenotypes. Reduction of Eiger in astrocytes, a population of cells with enriched Eiger expression [43], resulted in a significant reduction in sleep duration compared to genetic controls, while no effect was observed in flies with neuronal Eiger knock-down. In addition to identifying a role of Eiger in baseline sleep regulation, our loss-of function studies found that Eiger was required for experience-dependent increases in sleep following social enrichment. Further, we found that Eiger expression in astrocytes was required for the increase in sleep characteristic of social enrichment. Therefore Eiger signaling in astrocytes regulates sleep in the fly, revealing a novel mechanism for understanding how astrocytes contribute to sleep.

Neuronal expression of the Eiger receptor, Wengen, is necessary for a normal homeostatic response to sleep deprivation. RNAi knockdown of Wengen in neurons or astrocytes did not alter baseline sleep duration. However, the neuronal RNAi knockdown, but not the astrocyte-based RNAi knockdown, significantly reduced homeostatic sleep rebound following sleep deprivation. This suggests that the Wengen receptor may be required for cytokine dependent homeostatic sleep regulation and that astrocyte-neuron communication via Eiger/Wengen signaling plays an important role in sleep homeostasis, but not in regulating baseline sleep duration. Our data indicate that increases in sleep following sleep loss requires Wengen receptor signaling in neurons. This finding is supported by the finding that the human TNFα injections in neuronal expressing Wengen RNAi flies failed at increasing sleep duration. We hypothesize that different TNFα receptors may differentially mediate the regulation of baseline sleep duration and sleep homeostasis. Grindelwald, for example, is another *Drosophila* TNFα receptor superfamily member that has been shown to bind Eiger and whose function differs significantly from Wengen [44]. Therefore, downstream signaling from Eiger via Grindelwald in neurons and/or glia may explain this dissociation between Eiger signaling and baseline sleep duration versus its homeostatic regulation. Future studies are required to elucidate the signaling mechanisms of Eiger and Grindelwald from those between Eiger and Wengen. Such studies would additionally benefit from over-expression analysis and from injection of Eiger instead of TNFα.

Genetic background can often be sufficient to affect behaviors such as sleep. However, through the use of multiple experimental approaches to elucidate Eiger function, we gained confidence that the phenotypes described here are not merely artifacts of genetic background. This conclusion is supported by the use of two loss-of-function mutants, one RNAi line, social enrichment experiments, and injections of human TNFα. It is possible that the human TNFα injection studies may have resulted in peripheral activation of a TNFα superfamily receptor that regulates sleep duration. However, our experiments showing that neuronal expression of Wengen is required for human TNFα induced sleep increases rules that out as the sole explanation for our findings.

Few studies have investigated how glial-specific factors and mechanisms regulate sleep [45–47]. We previously identified the astrocyte-derived fatty acid-binding protein 7 (Fabp7) as an important regulator of sleep across diverse species [45]. Fabp7 deficiency in astrocytes decreased the activity NF-kB and TNFα following lipopolysaccharide stimulation [48], which promotes inflammatory cytokine production [49]. Since Fabp7 can influence both NF-kB and TNFα expression in astrocytes, this suggests that Fabp7 may be directing its effects on sleep through cytokine and/or inflammatory pathways. It is currently unknown whether NF-kB and TNFα can in turn affect Fabp7 expression. A reciprocal feedback loop involving astrocytes would provide a unique framework for differentiating baseline sleep regulation from sleep homeostatic processes.

## Materials and methods

### Fly stocks

Canton S, w^1118,^ UAS-Eiger RNAi, UAS-Wengen RNAi, Elav-Gal4, and Alrm-Gal4 stocks were all obtained from the Bloomington Drosophila Stock Center (Indiana University). EGR1 and EGR3 mutants on CS background were obtained from Dr. Masayuki Miura (University of Tokyo).

### Fly husbandry

Flies were cultured at 25°C, 60% humidity, maintained on a 12:12 Light:Dark cycle, on Nutri-fly Bloomington Formulation fly food (Genesee Scientific, San Diego, CA.). Newly eclosed virgin female flies were collected from culture vials daily under CO_2_ anesthesia and housed in groups of ~30 prior to experimentation.

### Intra-thoracic microinjections of TNFα

Adult 4–6 day old wild-type w^1118^, virgin, female flies were injected at ZT3 with 9.2nL of 1nM, 10nM, 100nM or 1000nM recombinant human TNFα (R&D Systems, Minneapolis, MN) in phosphate-buffered saline (PBS) using a glass pulled pipette and an automatic nanoliter injector (Drummond Scientific, Broomall, PA). Flies were injected under CO_2_ anesthesia in the ventrolateral surface of the fly thorax and immediately placed into the behavioral monitoring system for measuring sleep.

### Sleep analysis

Female flies 4–7 days after eclosion were used for all sleep studies. Flies were mouth aspirated into 5mm x 65mm (outside diameter x length) polycarbonate recording tubes (Trikinetics, Waltham, MA) with food (Bloomington Nutrifly formula) on one end and yarn plugs on the other. Sleep parameters were continuously evaluated using the Trikinetics *Drosophila* activity monitoring system (DAMS; Trikinetics, Waltham, MA) as described previously [24]. One acclimatization day was followed by two days of baseline sleep recording, one 12-hour sleep deprivation period during the dark period, or one 24-hour sleep deprivation period (see main text), and two full days of recovery sleep. Sleep deprivation was performed as previously reported [24] using a custom-built Sleep Nullifying APpartus (SNAP; Machine Shop, Washington University in Saint Louis). Sleep homeostasis was calculated for each individual as the ratio of minutes of sleep gained above baseline during recovery divided by minutes of sleep lost during sleep deprivation (min gained/min lost).

### Social enrichment

To standardize the environmental conditions during critical periods of brain development, virgin female flies were collected upon eclosion and maintained in same-sex vials containing ~30 flies for 2 days. This protocol keeps environmental conditions constant between subsequently isolated and enriched flies for the first 2 days of adult life. Three-day old flies were then divided into a socially isolated group, in which flies were individually housed in 65 mm glass tubes, and a socially enriched group, consisting of 50 female flies housed in a single vial as previously described [1]. After five days of social enrichment/isolation, flies were placed into clean 65 mm glass tubes and sleep was recorded for three days using the Trikinetics DAMS.

### Data analysis

Statistics were calculated using Graphpad Prism software. Student’s t-test, one-way ANOVA, two-way ANOVA, and Tukey post-hoc analysis were used for analyses. Sleep data were analyzed by averaging across multiple experiments. Flies that did not survive the entire experimental paradigm were removed from data analysis. The ActogramJ plugin for ImageJ was used to quantify circadian period and daily activity levels.

## Supporting information

S1 Fig Eiger mutants have a reduced homeostatic response to sleep loss. Following 12 hours of sleep deprivation in wild-type CS, EGR1, and EGR3 flies. CS flies recovered 40% (n = 46, SD = 43.29) of lost sleep in the 48 hours after sleep loss. The EGR1 (n = 24, M = 3.8, SD = 72.0), and EGR3 (n = 21, M = 9.7, SD = 66.2), flies showed a significantly reduced homeostatic response compared to CS flies (F(2,90) = 5.99, p = 0.0036). Asterisks indicate values that are significantly different (P < 0.05) from CS control flies as determined by Tukey’s Multiple Comparison Test.
(EPS)

S1 Data Figure data. (XLSX)
